# Examining the Range of Motion of the Cervical Spine: Utilising Different Bedside Instruments

**DOI:** 10.21315/mjms2021.28.2.9

**Published:** 2021-04-21

**Authors:** Aiman Asyraf Ahmad Sukari, Sarwinder Singh, Muhammad Hafiz Bohari, Zamzuri Idris, Abdul Rahman Izaini Ghani, Jafri Malin Abdullah

**Affiliations:** 1 Department of Neurosciences, School of Medical Sciences, Universiti Sains Malaysia, Kubang Kerian, Kelantan, Malaysia; 2 Hospital Universiti Sains Malaysia, Universiti Sains Malaysia, Kubang Kerian, Kelantan, Malaysia; 3 Neurosurgical Unit, Department of General Surgery, Hospital Tuanku Jaafar, Seremban, Negeri Sembilan, Malaysia; 4 Department of Neurosurgery, Hospital Kuala Lumpur, Kuala Lumpur, Malaysia; 5 Department of Neurosurgery, Hospital Queen Elizabeth, Kota Kinabalu, Sabah, Malaysia; 6 Neurology Unit, Department of Internal Medicine, School of Medical Sciences, Universiti Sains Malaysia, Kubang Kerian, Kelantan, Malaysia; 7 Brain and Behaviour Cluster, School of Medical Sciences, Universiti Sains Malaysia, Kubang Kerian, Kelantan, Malaysia

**Keywords:** examination technique, cervical range of motion, rotation, flexion

## Abstract

**Background:**

This paper outlines a summary of examination technique to identify the range of movement of the cervical spine. Due to common difficulties in obtaining tools for cervical examination within the district, a standardised compilation of easy-to-replicate examination techniques are provided using different tools.

**Methods:**

Bedside instruments that can be used includes a measuring tape, compass, goniometer, inclinometer and cervical range of motion (CROM) instrument.

**Discussion:**

Cervical flexion-extension, lateral flexion and rotation will be assessed with bedside instruments. This would aid in increasing accuracy and precision of objective measurement while conducting clinical examination to determine the cervical range of motion.

## Introduction

The function and significance of the spinal vertebrae cannot be overstated. Its roles include serving as anchor for limbs and the head, corralling the spinal cord, besides being a significant attachment to the rib cage and torso muscles. Hence deriving its common name in the literal and figurative sense, the backbone. The vast functions of the spine stems from changes of its structural anatomy across the vertebrae, giving rise to different functional anatomy and mobility properties (1). Thus, examining different parts along the vertebrae may provide different measurements and range of movement during clinical examination.

This paper aims to provide a brief overview of how to examine the cervical range of motion via easily available bedside instruments. Specifically, the goniometer, inclinometer and the cervical range of motion (CROM) instrument. However, this paper will not detail associated neurological examinations of cervical spine nerve roots or extremities, nor will it detail additional signs that may be elicited from the cervical spine examination. As such, manoeuvres like the Bakody’s sign and Jackson’s compression test shall not be mentioned.

A typical examination should always begin with a general inspection, followed by palpation, range of motion and special manoeuvres. This paper, however, will focus mainly on assessing the range of motion of the cervical spine. It is important to note that complete examination of the vertebrae should only be conducted when there is no evidence of acute fractures or ongoing instabilities of the spine. In case of presence of spinal fracture or instability, utmost caution should be exercised during spinal examination and movement, besides adhering to appropriate protocols relevant to such cases.

## Methods

No specific equipment is required during the general inspection and physical examination of the cervical spine. However, for the range of motion assessment, utilising certain tools allows for an objective and standardised assessment during follow-up. Bedside instruments that can be used may include a measuring tape, compass, draughtsman’s flexible ruler (2), finger-floor method, goniometer, inclinometer and CROM instrument (3). Given that the aim of this paper is specific to bedside instruments, adjuncts such as X-rays, imaging and motorised or electrical equipment like digital goniometers and inclinometers, shall not be mentioned further. A link demonstrating the examination technique using bedside instruments can be viewed at the following link: https://youtu.be/3_ztdQBQMTI.

## Cervical Flexion and Extension

Request for the patient to sit up straight on a chair with his/her thoracic spine positioned against the back of the chair, arms dangling to their sides and feet flat on the floor. Then, observe the patient from the side. This position can be considered as 0°. To assess cervical flexion, ask the patient to nod forward and bring their chin towards their chest. Normal cervical flexion is usually approximately 80º. To assess cervical extension, ask the patient to look upwards as far as possible, until full extension of the neck is achieved. Normal cervical extension is usually 50°. Total range of cervical motion from full flexion to full extension should be 130° (4). Nevertheless, it is possible to gauge if the patient has normal cervical flexion if he/she is able to touch his/her chest with their chin.

### Tape Measure

The lowest end of the sternal notch should be identified as the fixed point or reference point. Then, ask the patient to flex and extend their neck. Measure the distance between the reference point to chin during maximal flexion and extension (5).

### Inclinometer

Place the inclinometer device on the top of the patient’s head, along the sagittal plane and ensure the reading on the inclinometer is at 0°. Then, ask the patient to flex and extend the neck. Record readings of the inclinometer at each extreme of the motion (6).

### Goniometer

Using a goniometer, first place the axis of the goniometer over the external auditory meatus. Align the stationary arm vertically or perpendicular to the floor. Align the moving arm to the base of the nose. Note this as 0°. Then ask the patient to flex and extend his/her neck and record readings of the goniometer at each extreme of the motion. The axis should remain at the external auditory meatus and the stationary arm vertical to the floor, but the moving arm should be realigned following the base of the nose (7).

### CROM Instrument II

Place the CROM instrument II apparatus on the patient’s head and ensure it is fit securely by adjusting the strap. Note the reading of the goniometer on the side of the head, which should read at 0° at neutral position. Then, ask the patient to flex and extend his/her neck, at which point readings are recorded at each extreme of the motion (8).

### Cervical Lateral Flexion

Ask the patient to sit up straight in a chair with the thoracic spine positioned against the back of the chair, with arms dangling on the sides and feet flat on the floor. Instruct the patient to look straight ahead, ideally at a fixed point of eye level. Observe the patient from the front. Consider this the starting point (i.e. 0°). Ask the patient to tilt his/her head laterally to the left, without rotating the head, while his/her shoulders remains fix (i.e. bring ear as close as possible to the shoulders without lifting the shoulders). Examiner can assist in fixing the position of the shoulder’s by gently placing their hands onto the patient’s shoulders. Repeat the procedure for the opposite side and note the angle of flexion of the head. Normal flexion from starting point on either side is 45° and the total angle of maximal lateral head flexion should be 90° (4). Eyeballing is difficult, therefore the use of a goniometer or CROM instrument may aid in accurate measurement of the angle of head tilt.

### Tape Measure

The acromion process on each side should be identified as the fixed point or reference point. Then, ask the patient to flex his/her neck sideways. Measure the distance between the fixed point to the lowest point of the earlobe during maximal lateral flexion. Repeat the same for the contralateral side (5).

### Inclinometer

Place the inclinometer device on the top of the patient’s head, along the coronal plane and ensure reading on the inclinometer is at 0°. Then, ask the patient to flex his/her neck laterally and record the readings of the inclinometer at each extreme of the motion (6).

### Goniometer

Firstly, place the axis of the goniometer; over the spinous process of the C7 if examining from the back, or at the sternal notch if examining from the front. Align patient’s stationary arm along the imaginary line between the two acromion processes either vertically or perpendicular to the floor or horizontal and parallel to the floor. Align patient’s moving arm; over the external occipital protuberance if examining from posterior or along the centre of the patients’ nose if examining from anterior. Consider this position as 0°. Then, instruct patient to flex his/her neck laterally and record readings of the goniometer at each extreme of the motion. Ensure that the axis and the stationary arm remains fixed throughout the motion and adjust the moving arm accordingly (7).

### CROM Instrument II

Place the CROM instrument II apparatus on the patient’s head and ensure secure fit by adjusting the strap. Note the reading of the goniometer on the front of the head, which should be at 0° when in neutral position. Then, instruct patient to flex the neck laterally and record readings at each extreme of the motion (8).

## Cervical Rotation

Ask the patient to sit up straight in a chair with the thoracic spine positioned against the back of the chair with arms dangling on the sides and feet flat on the floor. Instruct the patient to look straight ahead, ideally at a fixed point of eye level. Observe the patient from above. Next, ask the patient to rotate his/her head to the left as far as possible without tilting or tipping his/her head. Stabilise the shoulders by lightly pressing onto them. Repeat the process for the opposite direction. Normal rotation is approximately 80° while the neck’s total angle of rotation is 160° (4). For a rough approximation, when observing from above, the patient’s chin should just be slightly anterior to the shoulder during maximal rotation on either side.

### Tape Measure

The acromion process on each side is identified as the fixed point or reference point. Then, ask the patient to turn his/her head to one side. Measure the distance between the reference point to the chin during maximal cervical rotation. Repeat the same for the contralateral side (5).

### Compass

A simple compass can be very useful in assessing the rotation angle of the head in the absence of a goniometer or the CROM instrument. Begin by placing the compass on the top of the patient’s head and ensure his/her face is pointing north, according to the direction of the compose needle. Then ask the patient to rotate his/her head while the stabilising his/her shoulder. Record the amount of change based on readings of the compass at maximal rotation on each side (8).

### Inclinometer

First, ask the patient to lie supine on the bed with his/her face looking straight upwards. Then, place the inclinometer device along his/her forehead, just above the eyebrows. Ensure the reading on the inclinometer is at 0°. Then ask the patient to turn his/her head sideways and record readings of the inclinometer at each extreme of the motion (6).

### Goniometer

First, place the axis of the goniometer from above over to the centre of the patients’ head. Align his/her stationary arm along an imaginary line between the two acromion processes. Align his/her moving arm at the tip of the nose. Then ask the patient to rotate his head. Record readings of the goniometer at each extreme of the motion. Ensure that the axis remains at the centre of the patient’s head, the stationary arm along the imaginary line of the two acromion processes, and the moving arm realigned following the tip of the nose (7).

### CROM Instrument II

Place the CROM instrument II apparatus on the patient’s head and ensure secure fit by adjusting the strap. Observe reading of the compass on the top of the patient’s head and adjust it accordingly until it is at 0°. Then, ask the patient to turn their head and record readings at each extreme of the motion (8).

## Discussion

Range of motion can be assessed by eyeballing. This however is a crude method of measurement and is fallible to observer bias. Besides, it is not an objective, accurate and reproducible measure of the spinal vertebrae motion.

A measuring tape is often easily accessible in clinics; it works by measuring changes in distance of cervical motion from a fixed body part. It is however not suitable for measuring the neck’s degree of motions. Nevertheless, it is a reliable method for clinical assessment of the cervical range of motion, with the exception of cervical extension (5). Though systemic review by de Koning et al. (9) remains doubtful of the reliability and validity of tape measure use due to lack of blinding in some studies.

An inclinometer allows for assessment of range of motion by measuring the difference between distinct angles of the head during motion. It is a non-complicated method that requires the use of just one hand. It also shows good reliability and intra-observer agreement of cervical assessment, but its validity remains in question (9).

A goniometer is used to measure angles of changes relevant to the bodily motions of the head, neck and back. It is often used to measure joint angles on limbs. The use of a goniometer allows for accurate measurement of the degree of movement of body joints. However the examiner is required to use both hands to adjust the arms of the goniometer during the examination, therefore unable to stabilise the neck of the patient during range of motion. There is however a crucial need to standardise the central axis of the joint for every movement, as changes in the axis by different examiners will lead to differing results. A systemic review maintains apprehension towards its reliability and validity (9).

The CROM instrument essentially encompasses goniometers and a compass that has been mounted on a rigid headgear. As such, its position remains consistent and allows for accurate measurement of the head and neck range of motion. The CROM instrument can be directly fixated to the patient’s head and body, thus eliminating examiner bias besides the need to define a fixated point on the body. It is also easy to administer upon set-up and does not require use of the hands. It does consume time to assemble and adjust based on the size of the patient’s head. It also costlier than all priorly mentioned instruments and there has been doubts surrounding its practicality for clinical use (10). The CROM instrument has been extensively studied and has shown good reliability and validity compared to other methods of examination (9).

Systemic reviews by de Koning et al. (9) and Yee Won et al. (11) recommends the use of inclinometer, goniometer and CROM instrument upon high rating on its clinometric properties and practicality. It is reliable method of measurement, far more than simple visual estimations.

## Conclusion

A consistent and standardised method should be implemented when evaluating range of motion of the cervical spine. That way, interobserver variability can be reduced and a reliable monitoring of progress or deterioration of cervical range of motion can be provided. Finally, it is crucial to conduct a complete upper limb neurological assessment, especially testing for brachial plexus to complete the cervical spine examination. Testing of the limbs could help elicit signs of insult to the spinal cord (e.g. Kernig’s and Brudzinski’s sign in meningitis). Examination techniques should also be modified according to the patient, and care must be provided at all times to avoid experience of unnecessary pain or duress towards the patient during vertebral examination.

## 
